# The Role of Artificial Intelligence and Machine Learning in Revolutionizing Probiotic Research

**DOI:** 10.3390/microorganisms14092103

**Published:** 2026-09-19

**Authors:** Reza Nori, Parvin Shariati

**Affiliations:** 1 Department of Systems Biotechnology, Institute of Industrial and Environmental Biotechnology, National Institute of Genetic Engineering and Biotechnology, Tehran P.O. Box 14965/161, Iran; rezanori277@gmail.com; 2 Department of Bioprocess Engineering, Institute of Industrial and Environmental Biotechnology, National Institute of Genetic Engineering and Biotechnology, Tehran P.O. Box 14965/161, Iran

**Keywords:** artificial intelligence, machine learning, probiotics, microbiome, personalized therapy, strain identification

## Abstract

The microbiome, as a vast and dynamic community of microbes, is now recognized as a key regulator of host physiology, profoundly influencing health and susceptibility to disease. Accordingly, probiotics are a mainstay of prevention and treatment. But the individual complexity and dynamic specificity of an individual’s microbiome make the previous “one-size-fits-all” research model completely invalid. This review systematically analyzes the applications of artificial intelligence (AI) and machine learning (ML) as transformative computational tools necessary to surmount these challenges. In this article, we detail how these computational methods have been applied throughout the entire research and development pathway of probiotics, including novel strain identification (through multi-omics analysis), formulation and production optimization, and elucidation of complex mechanisms of action in the host. Furthermore, we highlight the emerging frontier of personalized probiotic therapy, demonstrating how AI/ML can be utilized to predict treatment efficacy based on individual host data. The objective of this article is to provide a detailed discourse on the actual and prospective applications of AI and ML in this process, ultimately delineating their revolutionary potential to inform the design of the next generation of probiotics with unprecedented precision, efficacy, and sustainability.

## 1. Introduction

The interaction between microbes and their hosts plays a central role in biology and significantly influences health and disease across species [1]. Probiotics, defined as live microorganisms that, when administered in sufficient numbers, confer a health benefit on the host, are primarily beneficial bacteria of the genera Lactobacillus, Streptococcus, Enterococcus, and Bifidobacterium, and have evolved from traditional fermented foods to advanced dietary supplements and topical treatments [2,3].

The idea of applying microbial intervention to the promotion of human well-being extends as far back as the early 20th century, when Élie Metchnikoff noted that the longevity of Bulgarian peasants was associated with the consumption of fermented milk. The work on probiotics has continued to evolve from general-spectrum benefits to an emphasis on strain-specific benefits [4]. Probiotics have an appreciable effect on health by modulating intestinal microbiota, stimulating defense by the host, and influencing an array of physiological functions [5]. They are effective when treating conditions such as irritable bowel syndrome (IBS), constipation, and all sorts of diarrhea [6,7]. Their immunomodulation role is also appreciable where probiotics interact with the immune system, potentially preventing infection as well as immune-mediated allergic responses. The gut–brain axis work has also produced “psychobiotics,” showing promise where they are able to treat depression and anxiety by modulating the neuroactive metabolites as well as by influencing the inflammatory pathways [8,9,10,11].

Probiotics also have probable roles to play in metabolic health through regulating energy intake, modulating satiety hormones to combat obesity, including enhanced insulin sensitivity in type 2 diabetes. They also play their role in vaginal health through the maintenance of healthy microbiota [12]. Their uses include dermatology, where they have the capability to treat skin disorders like acne and eczema through the modulation of immune responses and barrier function. Some probiotics also serve to decrease dental issues. Their uses have also been noted among athletes, to complement the immune system as well as to aid improved absorption of nutrients [13,14].

Although promising, probiotic science is faced with the grim reality of the intrinsic complexity of biological systems. The human microbiome and specifically the gut microbiome are an intractably complex and dynamic system that harbors trillions of microbes with a combined genomic burden far in excess of that of the human host. Each individual’s microbiome is unique, influenced by genetics, diet, lifestyle, geography, and early-life events [15].

Artificial intelligence (AI) and machine learning (ML) have been disruptive technologies in the field of microbiome and probiotic research given their distinctive abilities to process and analyze large and complex data sets. These novel technologies enable the scientific community to decipher the complexities of microbial interactions, antedate the response to the host, optimize strains of probiotics, and thereby extract novel insight into the greatly interconnected relations descriptive of microbiomes. By surmounting the simplistic plane of correlations, AI and ML enable the conceptual development of predictive models capable of educating the development of next-generation probiotic therapeutics. This holistic review sweeps across present as well as future uses of AI and ML throughout the probiotic research and development pipeline [16,17,18]. This article examines how these computational methods are being used in the identification of novel probiotic strains, formulation and scaling up of their production, and elucidation of their mechanisms of action in hosts. The main focus is on the emerging field of personalized probiotic therapy, in which artificial intelligence and machine learning are used to predict the efficacy of probiotics based on individual host information. This article highlights the important role of artificial intelligence and machine learning in the rational design and creation of probiotics and their potential to increase precision, efficacy, and sustainability in the development of probiotic products [17,19].

## 2. Data and Computational Methodologies

The machine learning and artificial intelligence revolution in probiotic research is more than sophisticated algorithms; it is heavily dependent on the massive amounts of biological data that exist. Analytical and high-throughput sequencing technologies provide a previously unimagined window into the intricate microbial universe and the behavior of microbes upon interacting with hosts. The form of data generated and the AI/ML paradigms underlying their analysis need to be comprehended to fully appreciate their disruptive power on probiotic science [17,20].

### 2.1. High-Throughput Omics Data of Probiotic Science

Biological research generates massive amounts of “omics” data, which provide comprehensive information about different biological molecules within a system. These high-density data sets are the first fuel of AI/ML algorithms and drive discovery and analysis of probiotics. 

AI/ML in probiotic studies are focused on the massive scale and diversity of omics data, which are provided by two critical pathways: public data repositories and specialized clinical collections. The majority of the genomic and metagenomic data are downloaded from databases such as NCBI SRA and MGnify, which contain genome sequences of thousands of probiotic strains and hundreds of thousands of metagenomic samples of the gut microbiome, usually in the range of hundreds of terabytes in size. Transcriptomic and proteomic (gene expression) data are also downloaded from databases such as GEO or MetaboLights. In addition to these resources, in order to facilitate high precision for therapeutic use, many AI models rely on in-house clinical libraries that connect multi-omics data (e.g., metagenomics, metabolomics, and precise clinical metadata) from target patient populations (including hundreds of samples). This type of connection between sequencing data and accurate phenotypic outcomes provides quality-labeled data for the training of AI models committed to precision probiotic selection and host response prediction [17,21,22].

In predicting host health effects, complex models like Graph Neural Networks (GNNs) are increasingly utilized. GNNs are specifically configured to model the core functional modules of host–gut microbiota interactions by treating metabolites, genes, and immune cells as nodes, and their regulatory relationships as edges. The GNN’s parameters (e.g., number of layers, aggregation function) are tuned to discover non-linear patterns, allowing it to predict, for example, the up- or downregulation of specific host cytokine profiles (e.g., IL-10 or TNF-α) in response to a novel probiotic input [23,24,25]. 

Genomics is one of the largest categories, which describes the complete genetic makeup of organisms. Probiotic strain whole-genome sequencing provides precise identification of genes that encode for beneficial properties like adhesion, antimicrobial function, immunomodulation, or resistance to stress, allowing for strain classification and their evolutionary history [21,26].

Metagenomics of host microbiota constitutes direct sequencing of environmental material, e.g., feces, to reveal the entire genetic content of all microorganisms within the sample. This provides a comprehensive list of species and potential functions in the host gut. AI/ML would identify correlations between specific microbial gene profiles, presence of probiotic species, and resulting host health outcomes [27,28].

Going a step further than gene expression, transcriptomics examines the full repertoire of RNA transcripts that an organism or a population of cells produces. mRNA sequencing (RNA-Seq) is an example and can determine what genes a probiotic strain is expressing under certain conditions, e.g., during fermentation or in the gut environment. It also gives insight into host gene response to probiotic treatment, leading to great understanding of how they work. AI/ML in such cases can recognize gene expression signatures that have direct correlation with healthy probiotic action [29].

Proteomics and metabolomics are synergistic approaches that are critical to acquiring a profound understanding of probiotic activity. Proteomics is the high-throughput description of proteins, the cell’s “principal workhorses,” which provides an instantaneous snapshot of the operational molecular machinery. By examining the proteins that the probiotic itself or the host produces in response to it, researchers can track the functional status of the probiotic and the individual accomplishments of the probiotic in the host environment. AI/ML models are introduced here, as they have the ability to associate specific protein profiles with positive probiotic activity (e.g., anti-inflammatory activity) or predict a dramatic host physiological response. At the same time, metabolomics tracks all of the small-molecule metabolites (e.g., fatty acids, amino acids, and vitamins) in a biological sample. Probiotics create a vast inventory of these metabolites, a large number of which are bioactive, including significant signaling molecules such as short-chain fatty acids. This approach enables the identification of such bioactive compounds and comprehending their role in probiotic function. By monitoring and studying the changes in host metabolite profiles upon consumption of probiotics, AI/ML is able to infer the specific host pathways affected by the probiotics and aid in the identification of new efficacy biomarkers [21,30,31].

Phenomics involves the high-throughput analysis of quantitative organism traits, or phenotypes, and, for probiotics, it brings about the quantitative assessment of several critical performance attributes; these assessments include stress resistance (such as measuring growth rates under low pH or bile salts), formulation stability (by quantifying viability across various formulations), production levels of key metabolites, and adhesion properties to host surfaces or cells. High-throughput robotic platforms generate enormous phenomic data sets, making them ideal for assessment by AI/ML models. These models play a vital role because they can provide precise predictions of optimal growth conditions, calculations of formulation stability, and forecasts of overall strain performance, ultimately making the integration of AI/ML with phenomics dramatically improve the efficiency and speed of the probiotic development process [32,33].

### 2.2. Principal AI and ML Paradigms

AI and ML provide the analytical power for producing valid conclusions from the vast omics and phenomics data [34,35].

ML techniques, essential for the deduction of meaningful information from complex biological data, essentially include two broad categories: supervised and unsupervised learning [36]. Supervised learning constructs models based on labeled datasets where the desired output is known and allows them to predict accurately on new, unseen data. In this framework, classification models are used where the output is a discrete category, as in categorizing a new microbial isolate as either “probiotic” or “non-probiotic” based on its genomic characteristics, or predicting whether a patient will be a “responder” or “non-responder” to a specific probiotic therapy [17,37,38]. 

The application of supervised classification models in probiotic discovery is a technical and rigorous approach that begins with the generation of advanced screening sets. In this process, raw omics data (such as genomic sequences) are transformed into quantitative inputs (such as gene abundance) using feature engineering. Models such as SVM or Gradient Boosting Classifier (GBC) are then trained to classify a new strain as “probiotic” or “non-probiotic” based on these features. The key technical advantage of this approach is the feature interpretability of models such as GBC and Random Forest, which can identify genomic features that are the strongest predictors of probiotic performance. This capability goes beyond mere prediction and greatly reduces the reliance on costly laboratory screening by providing mechanistic insight (insight mechanism) and computationally filtering candidates. For example, Sun et al. (2022) developed the iProbiotics platform using k-mer composition analysis of whole genome sequences and the SVM algorithm [39]. The model has a prediction accuracy of 9.77% and an AUC of 98.00% [17,39,40].

Conversely, regression models are employed when the output is a continuous value, such as predicting the optimal growth temperature for a particular probiotic strain or predicting the predicted reduction in IBS symptoms based on an individual’s personal microbiome composition [41].

Alternatively, unsupervised learning processes unlabeled data to identify hidden patterns or underlying structure without prior knowledge of the outcomes. Clustering algorithms, a core component of unsupervised learning, are used to consolidate similar data points into clusters. This is invaluable in microbiome research for activities like discovering special “enterotypes” in the human gut microbiome—natural groupings of microbial assemblages—or clustering probiotic strains based on their functional similarities from various omics data sets. In addition, dimensionality reduction aims to simplify complex datasets by reducing the number of variables without compromising the most critical information. The process is highly important when it comes to establishing the most influential microbial species or host genes that contribute immensely towards a specific health outcome, thereby making the high-dimensional biological data more interpretable, further analyzable, and easier to visualize [42,43,44].

Deep learning (DL) is a strong branch of ML, leveraging artificial neural networks with numerous layers—most often known as deep networks—to learn sophisticated patterns directly from unprocessed, raw data [45]. This renders DL particularly adapted to handling large datasets like raw sequencing reads, biological images, or time-series microbiome data [46]. DL models, such as Convolutional Neural Networks (CNNs) for sequence analysis or Recurrent Neural Networks (RNNs) for time-series data, are well-suited to identify subtle, non-linear connections that more traditional ML methods would fail to recognize. This capacity leads to greater accuracy when, for example, estimating the efficacy of probiotics or analyzing the subtleties of host-microbe interactions [46].

Reinforcement learning (RL) is another standalone ML framework where an intelligent agent learns to make the optimal possible choice by interacting with an environment in order to maximize a reward provided. Though not yet universally seen within direct probiotic applications, RL is highly capable of maximizing dynamic biological systems. For example, imagine a bioreactor is being controlled by an RL agent: it could learn and execute the optimal feeding regimes or alter temperature settings in real time, constantly trying actions and observing the impact they have on probiotic yield or viability as conditions dynamically shift. Adaptive learning supports extremely automated and effective process optimization [47,48,49].

Lastly, network analysis, through graph theory, is a strong method to model and understand the complexity of biological interactions. This method allows probiotic scientists to map the complex interactions between the host and microbes or microbes and microbes within the gut microbiota such that species or molecules are nodes and interactions between them edges. Network analysis can identify keystone species (efficient species) in the whole microbial community whose presence or absence can alter the balance of the community and predict how a specific probiotic transforms the underlying networks of interactions and predicts stability, resilience, or specific functional implications for health benefits [50,51,52].

## 3. AI/ML in Probiotic Strain Identification and Discovery

The search for novel probiotic strains with improved benefits is a pillar of contemporary microbiome research. Such work has in the past been slow and cumbersome, depending on techniques that are based on culturing bacteria and phenotypic screening [53]. Such traditional techniques tend to lose sight of the large array of microbes we cannot grow in the laboratory. Now, AI and ML are transforming probiotic discovery by performing it much faster by enabling us to analyze complicated biological data at high speed. This speeds up the identification of potential new candidates and enables us to know what they can do.

### 3.1. Discovery of New Probiotics

For starting the discovery of new probiotics, we need to look broadly in the microbial universe. Artificial intelligence technologies are most suited to search through various sources of data to find such gems. Strong among them is the application of Metagenomic Sequencing of Environmental and Host-Associated Microbiomes. Most microbes that live in the soil, water, or human intestine cannot be grown in a laboratory. Metagenomics enables researchers to sequence all of the DNA directly from the samples, giving us an entire genetic catalog of the entire microbial community. AI and ML programs would then browse through these massive datasets in search of new genes or even entire genomes of previously unknown bacteria that could possess the trait of probiotics. This is usually through a suite of tools that align sequences, predict genes, and describe what capabilities a gene can offer (such as metabolic functions, antibiotic function, or adhesion function) based solely on its genetic sequence. For instance, ML models can be trained on identified probiotic genomes in order to recognize patterns related to processes like bile salt hydrolysis or SCFA production, even among organisms that we have yet to successfully culture [39,54,55].

Outside of new environments, there are large collections of known microbial species. AI/ML can perform Large-scale Genomic Analysis of Known Microbial Collections to identify probiotic traits. By surveying thousands of bacterial genomes, machine learning algorithms can find genetic markers, like individual genes for adhesins or exopolysaccharides that allow bacteria to stick to surfaces, or bacteriocins that are toxic to other microbes, which are known to be linked with probiotic advantages [17,39,56]. This will subsequently enable us to rapidly re-evaluate and categorize current strains, which can possibly unlock new applications for microbes we have already investigated for other applications. Finally, it is hoped that one can predict what a microbe can do from its genetic blueprint itself. Deep learning algorithms, such as Convolutional Neural Networks (CNNs) or Recurrent Neural Networks (RNNss), are able to learn complex patterns from raw DNA or protein sequences directly [57,58]. For example, they are able to predict whether a bacterium possesses genes that allow it to stick to the lining of the gut, secrete antimicrobial peptides to block pathogens, or regulate stress (which is critical for survival in the gut). By examining these genetic markers, AI helps us prioritize the top candidate strains with the greatest prospects for subsequent laboratory study, greatly enhancing the search for new probiotics [59,60].

### 3.2. Probiotic Trait Prediction Using Predictive Modeling

Once scientists identify promising probiotic candidates through genomic sequencing, AI/ML steps in to supercharge the work of phenotypic screening. This brings us from laborious, mechanistic laboratory tests to quick, forward-looking, and high-throughput systems [17].

One of the significant methods that happens is via Machine Learning Models that Classify Strains According to Desired Traits. How effective a probiotic is will depend on numerous characteristics, such as how well it tolerates extreme conditions in the gut (acid, bile salts), how well it adheres to cells lining the intestines, and how strong an effect it has on the immune system. Supervised machine learning algorithms—e.g., Support Vector Machines, Random Forests, or Gradient Boosting Machines—can be trained on experimental results from strains previously tested for such characteristics. Then, using these models, they learn to translate particular protein or genetic features into these observable traits. This makes prediction of new strains possible in silico. Essentially, we can predict if a new strain will be acid-tolerant or produce a particular immune-modulating metabolite even before we have invested the cost and time of laboratory trials [61,62].

Moreover, High-Throughput Phenotyping Coupled with ML greatly streamlines the screening of large libraries of microbes. Existing robotic platforms can quickly measure many attributes across thousands of microbial strains. These create vast datasets, so-called phenomic datasets, holding data like growth curves at different pH, how they biofilm, or what metabolites they excrete. There is a necessity for AI/ML algorithms to sift through the vast data. Unsupervised learning techniques, like clustering, can classify strains that have similar performance and help us detect patterns that we might otherwise miss. Concurrently, supervised learning can predict the performance of unseen strains based on their genetics and the known performance of similar strains. This powerful synergy significantly enhances the entire process of screening to the point where scientists can quickly identify top-performing candidates from large and diverse collections of microbes [63,64].

In an applied study, an Active ML approach was used to predict the effect of 111 pharmaceutical excipients on the growth of the probiotic Lactobacillus paracasei, using raw Isothermal Microcalorimetry data. The method specifically focuses on Uncertainty Sampling to maximize laboratory efficiency [33].

### 3.3. Identifying Functional Genes and Metabolites

The real strength of a probiotic is typically in the specific genes it carries or the metabolites it produces, rather than its species label. AI and ML capabilities are now allowing us to penetrate so much deeper into such functional details [65].

One such developing capability is AI-Driven Annotation of Novel Genes Responsible for Probiotic Effects. Many mechanisms through which probiotics act include specific gene products. Traditionally, gene functions are determined by comparison to known genes. But now, using the power of AI, especially deep learning, we can predict the functions of new or hypothetical genes even without seeing the sequence pattern, protein domains, or how they look next to other genes in the genome [66]. This allows researchers to pinpoint new, previously unknown genes, which may be new enzymes, regulatory proteins, or surface adhesins that create beneficial traits for the probiotic. This is vital for the “why” and “how” of the probiotic effects, making it crucial in genetically modified next-generation probiotics design [67]. 

For example, for the functional analysis of probiotic strains of *Limosilactobacillus reuteri*, ML-based bioinformatics tools such as eggnog-Mapper were used for genome-wide functional annotation. These computational tools were able to automatically generate specific genes that rapidly identify and characterize beneficial probiotic traits, such as genes for bacteriocins (antimicrobial compounds) and genes associated with resistance to harsh conditions. This mapping of functional layers of the genome gives us an understanding of the mechanism of action of probiotics (i.e., what metabolites are produced) and paves the way for the development of targeted engineered probiotics with the ability to synthesize more bioactive compounds [68,69].

**Figure 1:**
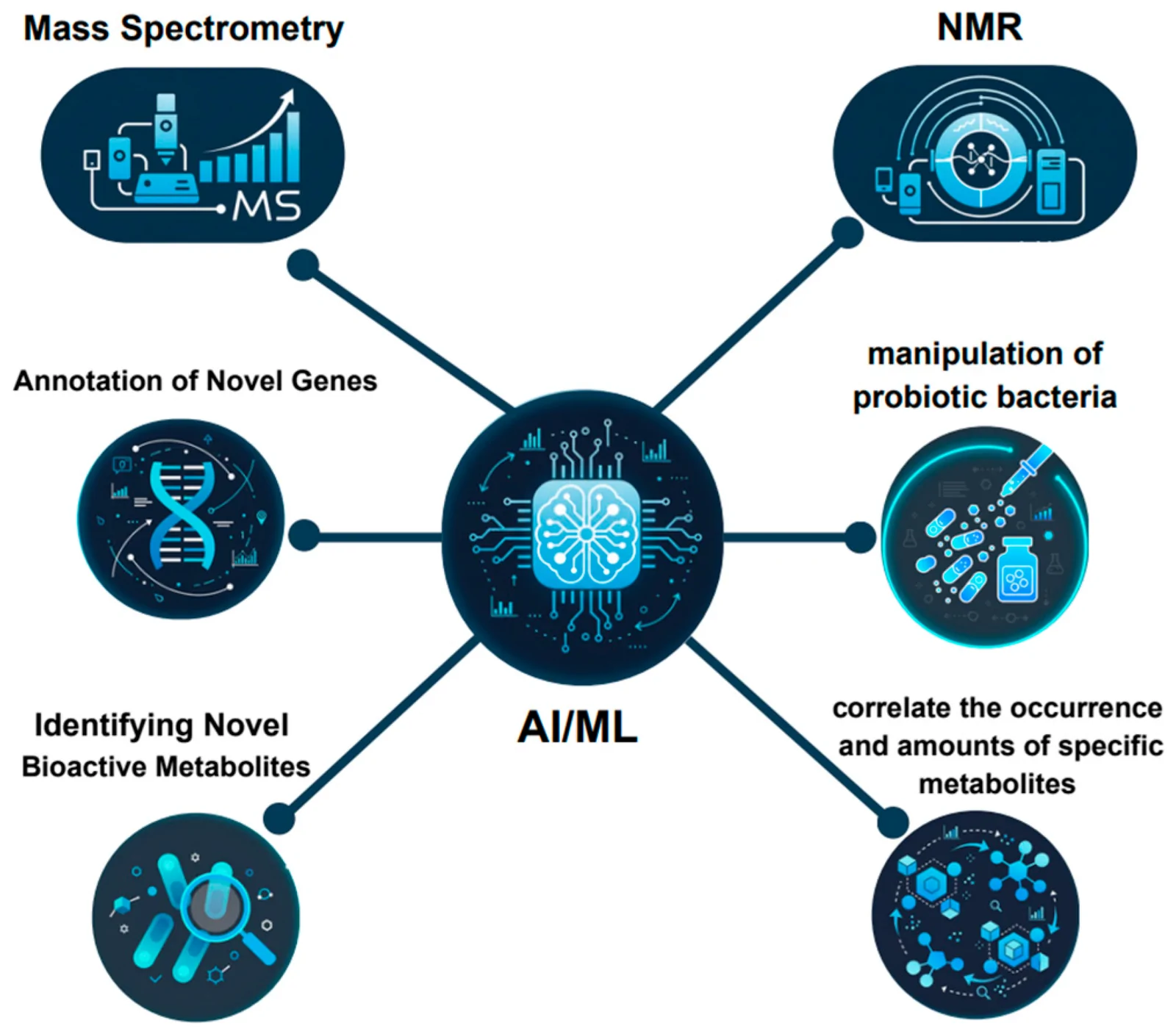
AI/ML-driven functional deep dive into probiotics.

Moreover, ML is robust for Identifying Novel Bioactive Metabolites Produced by Probiotics. Probiotics work through many effects by producing bioactive compounds such as short-chain fatty acids, polyamines, vitamins, or bacteriocins. Metabolomics, which yields complex information, makes it difficult to identify and quantitate all these molecules. Machine learning techniques, including chemometrics and deep learning, may employ data from techniques like mass spectrometry and NMR to identify and characterize these new metabolites. In addition, ML can correlate the occurrence and amounts of specific metabolites with probiotic activities observed in experiments or animal models. This enables us to uncover the “active ingredients” of a probiotic, paving the way for the development of postbiotic medicines or the manipulation of probiotic bacteria to produce more of these active molecules [41,70,71]. Figure 1 shows an in-depth look at AI/ML based performance in probiotics.

A summary of AI/ML in Probiotic Strain Identification and Discovery content is presented in Table 1.

## 4. AI/ML in Optimizing Probiotic Formulation and Production

One significant problem facing probiotic products is ensuring cell viability from the moment they are produced and stored until they pass through the hostile gastrointestinal tract. AI and ML, however, bring with them great tools to design much more stable products and new delivery systems that overcome these issues [72].

### 4.1. Formulation Optimization, Stability Prediction, and Targeted Delivery System Design

In the domain of formulation and production optimization, two distinct pathways exist. The established methodologies primarily utilize Artificial Neural Network (ANN) modeling or Fuzzy Logic to optimize static fermentation conditions (e.g., temperature, pH, and substrate concentration) aimed at maximizing cell yield. These models are trained on historical batch experiments and are highly effective for refining existing processes. However, theoretical possibilities are moving toward the adoption of reinforcement learning (RL) to create autonomous bioreactor systems. These RL systems can dynamically and in real-time adjust production parameters to maintain maximum cell viability and stability under shifting conditions, which is still in the early stages of industrial-scale development [25,48,73].

AI plays a vital role in predicting better encapsulating materials and drying technologies. Probiotic cells are inherently sensitive to environmental stresses like oxygen, humidity, and temperature. Techniques like encapsulation (e.g., alginate, chitosan, or whey protein) and dehydration techniques (like lyophilization or spray drying) are critical in protecting them. Material selection and drying conditions can significantly impact cell viability. AI/ML models trained on extensive experimental data from various encapsulation and drying runs can predict the optimal combination of encapsulation material blend, coating thicknesses, and drying conditions—temperature, duration, pressure, and excipients. This can maximize cell viability and ensure long-term stability, allowing rational formulation design of stable formulations tailored for specific probiotic strains and product needs.

ML tools, utilizing data from membrane fluidity assays, stress-induced gene expression profiles, and cell wall composition analysis, can forecast the Post-Processing Viability (PPV). For instance, a Random Forest classifier can be trained on a dataset integrating the upregulation of heat shock proteins (HSPs) (from transcriptomics) and the concentration of membrane unsaturated fatty acids (from fluidity assays) across 50 different Lactobacillus strains [74,75,76].

Machine learning models can also efficiently assess stability across various storage conditions. Probiotic goods are subject to varying environmental conditions during their journey around the world. ML models, by learning from real-time and accelerated stability data, would be able to forecast the shelf-life and the probability of decline in probiotic viability at various temperatures, humidities, and packaging styles. This provides valuable information for the setting of appropriate expiration dates, the design of best-in-class packages, and the enhancement of cold chain logistics, all aimed at maintaining product quality from point of manufacture to point of consumer consumption [77,78,79].

Aside from merely protecting probiotics, AI/ML is also used in computational design of new delivery systems for targeted release. For probiotics to be optimally effective, they sometimes need to withstand the acidic environment of the stomach and be delivered at very precise locations in the gut, e.g., the colon. AI/ML can in theory design high-level delivery vehicles like pH-sensitive coatings or microgels to deliver probiotics precisely where they are needed. ML algorithms analyze various polymer properties, degradation rates, and in vitro release profiles to predict the optimal composition and structure of such delivery systems. This enhances the viability of probiotics and considerably enhances their effectiveness in vivo while making them effective enough to perform the desired activity [80,81].

### 4.2. Quality Control and Contaminant Detection

Ensuring probiotic product purity and quality is absolutely crucial to product performance and consumer safety. AI and ML provide powerful, cutting-edge capabilities for fast and sound quality control from beginning to end during the production process [67,82]. One of the main applications is the use of machine vision and deep learning for automated quality inspection. During production, factories and companies tend to use visual inspection to detect foreign contaminants, abnormal product appearance, or packaging defects. However, deep learning architectures like Convolutional Neural Networks (CNNs) can be trained on huge collections of images of probiotic products. After being trained, these machine vision systems can detect problems like abnormal color, small particles, or packaging defects that can signal contamination or degradation in a quick and precise way. This process mechanizes the process of quality control, which makes it much faster and more precise than traditional manual checks [83,84].

Beyond the unaided eye, AI also powers microbial community analysis to detect contaminants along production lines. Determining the genetic purity of the probiotic strain and the absence of unwanted microbial contaminants is crucial. High-throughput sequencing methods like 16S rRNA gene sequencing or shotgun metagenomics can produce very sensitive profiles of the microbial communities in direct line samples from the production line. AI/ML algorithms are then used to scan these complex sequencing datasets to identify off-target microbes or even potential pathogens if they are present in extremely low levels. Through creating baseline profiles of what the microbial communities should be like at every stage of production, AI will be able to rapidly detect if there is any deviation, creating an early warning system against contamination. Moreover, ML is able to track changes in microbial populations with the passage of time and calculate potential risks of contamination against environmental parameters or process differences. This highly sensitive detection capability is of great value for maintaining maximum quality control in the manufacture of probiotics [85,86].

## 5. AI/ML for Understanding Probiotic Mechanisms of Action

As their health impacts become increasingly well established, it is usually not known through what molecular and cellular mechanisms they are operating. In order to enhance the design of effective and targeted interventions, knowledge of these complex interactions between the probiotic strains, host, and resident microbiota is fundamental. Machine learning and artificial intelligence are also increasingly proving their value in deconstructing such complex mechanisms, moving beyond mere correlations to identify causality in determining causal relationships in determining the complex effects of probiotics on host physiology [87].

Understanding the complex mechanisms of action of probiotics also follows this dual path. The proven application of AI involves using network analysis and feature selection algorithms on host transcriptomics or protein interaction data to target and identify key metabolic pathways and molecular checkpoints affected by probiotics (e.g., inflammatory pathways). These models are critical for dimensionality reduction in large omics datasets. However, the future potential lies in the development of AI/ML-driven Digital Gut Twins. These digital twins would be capable of systemically and holistically simulating the effects of various probiotics on entire physiological axes (e.g., the gut–brain axis), reducing the need for initial animal and clinical trials. Successful implementation of such large-scale human body modeling, however, remains a substantial challenge [88,89,90].

### 5.1. Deconstructing the Host-Microbe and Microbe-Microbe Interactions

The gut is an active and dynamic space where probiotics engage in an active dialogue with the host as well as the resident microbial community. Deciphering these interactions is of paramount importance to unlocking the whole potential of probiotic treatments.

One of the powerful ways of dealing with such intricate interactions is network analysis and graph theory. The gut microbiome can merely be thought of as a gigantic, intricate network of microbial species, their genes, and their metabolic products. Network analysis, a robust tool that has been evolved based on graph theory, allows researchers to represent such interactions [91]. In these models, individual microbial species, genes, or metabolites can be considered as nodes, and the interactions between them—i.e., competition for substrates, metabolic cross-feeding, or inhibition—are considered as edges. AI/ML algorithms can handle large-scale metagenomic, metatranscriptomic, and metabolomic data to create such complex networks. If a probiotic strain is introduced into an in vivo or simulated environment, AI can predict how it will integrate into the current network, what species it would be predicted to interact with, and how these interactions would affect the overall community structure and function [17]. This powerful approach is required to identify “Pivotal Microbes”—ones that are present or absent in a manner that disproportionately impacts the entire community—and for having knowledge of how probiotics can nimbly lead the ecosystem to a desired healthy equilibrium [92]. For example, ML might demonstrate that a given *Lactobacillus* strain consistently wins against a recognized pathogen by producing certain antimicrobial compounds or that it promotes the outgrowth of health-enhancing butyrate producers through metabolic cross-feeding [93].

In addition to simply charting these interactions, machine learning is also very good at delineating the most impactful participants in the microbial community, commonly known as “keystone species,” and making clear their unique interactions with probiotics. Computational tools such as Random Forests or Gradient Boosting Machines can identify particular microbial species or functional modules whose presence or activity closely tracks specific health outcomes or probiotic intervention responses. For instance, if a probiotic consistently improves metabolic markers, ML can identify particular changes in the composition or function of the resident microbiota that are associated with the improvement. This allows researchers to understand not only the direct impact of the probiotic but also how it indirectly affects the larger microbial network to produce its beneficial effect on host physiology [94].

### 5.2. Predicting Impact on Host Physiology

Probiotics achieve their health benefits through the modulation of many host physiological pathways, and AI/ML provides robust tools for connecting these microbial processes to host responses directly. The actual effect of a probiotic is observed by how it affects the host, which mandates integrating information across many layers of biology.

AI/ML models can successfully incorporate multi-omics data—combining complex microbiome profiles (defining microbial composition), host transcriptomics (defining active host genes), and host metabolomics (defining active host metabolites produced or consumed). By training on such combined datasets from clinical trials or in vivo experiments, advanced ML algorithms, including deep learning, can build strong predictive models of probiotic effects on host pathways. For instance, an ML model might be trained to predict that a specific probiotic consistently downregulates host inflammatory cascades or increases production of specific neurotransmitters through alterations in gut microbial metabolism. Such capability is crucial in understanding the mechanism of the probiotic in the case of diseases like inflammatory bowel disease or anxiety [17,41,95,96].

Additionally, AI is critical in identifying specific microbial metabolites or effector molecules responsible for observed health impacts. Most of the probiotic effect is exerted by their bioactive compounds, but it is challenging to identify these specific compounds within the protean complexity of a metabolome. AI/ML, and applying techniques like feature selection and correlation analysis to metabolomics data, can determine which microbial metabolites are consistently correlated with health benefit outputs. For example, if a probiotic improves obesity, ML can detect a novel short-chain fatty acid or one bile acid change that is directly linked to improved metabolic markers. Aside from metabolites, AI can also help predict novel effector molecules such as secreted proteins, bacteriocins, or immunomodulatory polysaccharides produced by probiotics through examining their genomic and proteomic data. Such intensive gastronomical data analysis allows us to identify insights into the exact “language” by which probiotics talk and influence the host [97,98].

### 5.3. Modeling Microbiome Dynamics Under Probiotic Intervention

The microbiome of the gut is not in a static state; it is an active system that is responding at any given time to diet, environment, and treatments such as probiotics. AI and ML prove extremely useful here, capable of modeling these complex dynamics and providing critical insights into probiotic establishment and persistence [99].

A compelling use case is using Deep Learning to model Microbiome Composition and Function Changes After Probiotic Intervention. For lasting efficacy of a probiotic, we ought to know its integration and restructuring of the inhabitant microbiome. Deep learning models like Recurrent Neural Networks (RNNs) or Long Short-Term Memory (LSTM) networks even analyze time-series metagenomic data that was gathered prior to, during, and following probiotic intake. These models are learning the complex, non-linear dynamics of microbial populations and are able to forecast, for instance, how a particular probiotic will alter the relative abundance of other species, modify functional gene sets, or affect metabolic pathways over time. This predictive capability allows researchers to more fully comprehend engraftment of probiotics (can the probiotic engraft in the gut) and how it can promote stable, beneficial changes in the resident microbiota [19,100,101].

In addition, AI/ML is instrumental in the process of forecasting the resilience and engraftment of probiotic strains within varied host microbiomes. It is well known that not all probiotics set themselves up well in everyone; their effectiveness significantly depends on the existing specific composition and functioning of the host microbiome. Machine learning programs can predict a host’s viability (survival and persistence of a probiotic strain within a competitive environment) and probable engraftment of a probiotic strain into a large population of host microbiomes. As training instances, ML can use baseline microbiome data, diet, and rates of clinical trial engraftment to determine which specific microbiome features of the host facilitate or inhibit probiotic colonization. This is the key to personalized probiotic guidance, ensuring that the chosen strain has the best chance of establishing itself and producing its wished-for effects on an individual [17]. Table 2 shows the main applications of AI and ML in the field of probiotics.

## 6. AI/ML in Personalized Probiotic Therapy and Efficacy Prediction

Natural variability of the human microbiome, as well as lifestyle, diet, genetic susceptibility, and other variables, means that a “one-size-fits-all” probiotic approach frequently produces uninterpretable results. The future of probiotic therapy is personalization, where personalized strains or blends are matched to an individual’s unique biological signature. AI and ML, the twin drivers of such a revolution, will fuel the unification of big data for predictive efficacy, personalized intervention, and reshaping clinical practice [60,102,103].

Personalized probiotic therapy will be based on the total profiling of an individual’s individual biology. To this end, AI/ML is the analytical bridge required, inasmuch as it is capable of bringing different biological data together in harmony with actionable, individualized recommendations for a probiotic [103,104,105].

Machine learning algorithms are optimally positioned to integrate a very wide array of disparate data, such as host genomic information, exhaustive gut microbiome snapshots, precise dietary histories, and lifestyle variables. Integrated together, they create a person’s distinctive biological geography. For instance, host genomic information can determine genetic predisposition to modulate gut immunity or nutrient metabolism. Gut microbiome composition, which is derived from advanced sequencing techniques like 16S rRNA gene sequencing or shotgun metagenomics, captures species makeup and functional potential. Further, precise records of eating habits (e.g., fiber intake, fat intake, fermented food intake) and other life habits (e.g., exercise frequency, perceived stress, medication use) are necessary inputs. Methods such as Random Forests, Gradient Boosting Machines, or Deep Neural Networks can pick up intricate, non-linear relationships between these diverse inputs and the health status or susceptibility to disease of a person. An example with practical application might be an ML system learning that a particular Bifidobacterium species is extremely effective at reducing bloating in patients with a particular genetic polymorphism of an enzyme that digests food, especially when combined with a diet rich in particular prebiotics [106,107,108,109].

The ultimate aim of synthesizing this multi-modal data is to go beyond generic recommendations and enable the generation of tailored probiotic prescriptions based on efficacy predictions for specific conditions. Once such an ML model has mastered such high-level relationships, it can calculate the likely effectiveness of a specific probiotic strain or designed consortia for a given patient with a given health condition, such as IBS, depression, or metabolic syndrome. For instance, a doctor could input a patient’s microbiome composition, gene markers, and dietary patterns into an AI platform. The system would then apply its vast learned knowledge base to recommend, “Based on your personal profile, *Lactobacillus plantarum* 299v, with a daily dose of resistant starch, is most likely to benefit your IBS symptoms,” well beyond the general “take any probiotic”. Highly specific and evidence-based, this approach significantly enhances the probability of a therapeutic outcome, effectively converting probiotics from general supplements into precision therapeutics [110,111].

## 7. The Role of AI/ML in Developing Next-Generation Probiotics

The applications of AI and machine learning extend far beyond the optimization of current probiotic strains; they are actually transforming the very fabric of probiotic development itself. By empowering never-before-possible predictive powers and automation possibilities, AI/ML is at the forefront of designing, testing, and delivering next-generation probiotics—strains optimized for improved efficacy, targeted action, and novel therapeutic applications. This is not a question of simply selecting useful microbes, but of rationally designing them [19,41].

### 7.1. Rational Design of Genetically Engineered Probiotics

Genetic engineering holds the promise to bestow probiotic strains with completely new capabilities or greatly enhance present ones, rendering them exponentially more efficient and effective in their therapeutic applications. AI/ML is crucial to this rational design process, much as it transformed it into a focused, knowledge-driven approach from its arduous trial-and-error predecessor [112].

One of these broad areas includes the AI-Driven Design of Novel Metabolic Pathways in Probiotic Strains for Enhanced Functionality. Just imagine a probiotic that not just inhabits the gut but actually produces a specific therapeutic molecule in situ. AI makes this achievable. Through computations from enormous databases containing information regarding metabolic reactions, enzyme kinetics, and microbial genomics, AI systems can computationally design entirely new metabolic pathways that may not necessarily exist naturally in a specific probiotic strain. For instance, in the case of a probiotic that seeks to overcome some deficiency, e.g., vitamin deficiency, AI could find and recommend the precise genetic changes (i.e., those specific enzymes and their respective encoding genes) which can be utilized to introduce or augment its production directly within the probiotic bacterium. Similarly, AI can design specific nutrient synthesis routes, cost-effective detoxification of harmful chemicals (e.g., oxalate in individuals with kidney stones), or programmed production of immunomodulatory metabolites. By this computational route design, not only is the introduced modification metabolically acceptable but also perfectly integrated into the host bacterium’s own metabolism in such a way that it maximizes the desired compound without incurring any metabolic burden on the cell [66,113,114].

Furthermore, machine learning is revolutionizing CRISPR-Based Genome Editing Strategies in Probiotics optimization. Precise genetic alteration, especially when introducing sophisticated pathways or executing many genomic modifications, heavily depends on advanced tools like CRISPR-Cas systems. Nevertheless, designing highly effective guide RNAs and predicting likely off-target effects precisely may be problematic. Machine learning algorithms can be tuned with experimental data from numerous CRISPR edit outcomes to enhance guide RNA design with forecasts of the most targeted and efficient sequences for gene insertions, deletions, or single-nucleotide polymorphisms in probiotic genomes. Above all, ML can also predict probable off-target edit sites, allowing researchers to choose strategies that prevent undesired genomic alterations. This accelerates the often time-consuming process of creating genetically engineered probiotic strains, making complex genetic modifications much more predictable, reliable, and safe [115,116,117,118].

### 7.2. AI Design of Synbiotics and Postbiotics

Together with individual probiotic strains, AI is enhancing the design of more sophisticated microbial therapies that leverage the synergistic power of combined beneficial components. This moves us closer to more synergistic and holistic approaches to gut health and beyond.

One of the most important uses is how AI may be utilized to identify the best synbiotics, or prebiotic/probiotic combinations. Synbiotics, or prebiotic/probiotic combinations that are synergistic for the benefit of the host, are a very effective therapy. The issue is, however, that identifying actually synergistic mixes out of the infinite set of possible mixes is very difficult. AI models are particularly designed to analyze high-dimensional data sets derived from in vivo models, in vitro co-culture experiments, and clinical trials (multi-omics data from probiotic as well as prebiotic components). This allows AI to make the optimal combination. For example, machine learning can predict which specific prebiotic (e.g., a specific fiber or oligosaccharide) is most conducive to the growth, viability, and functional activity (e.g., the generation of short-chain fatty acids) of a chosen probiotic strain in the ever-changing gut environment. This enables the rational optimization of highly effective synbiotics that achieve maximum therapeutic response [17,119,120].

Postbiotics are the non-viable microorganisms and/or fractions that confer a beneficial health effect to the host, such as desired microbial metabolites, cell wall components of the cell, or secreted proteins. Probiotics rely on live organisms, while postbiotics possess numerous benefits such as stability, safety (because they contain no live microbes), and convenience of storage. Machine learning has a two-fold role in this context: it helps identify new bioactive postbiotics by analyzing metabolomics information of probiotic fermentations and correlating specific compounds with determined health effects in in vitro experiments or animal models. Once a potential postbiotic is identified, ML algorithms can be used to design fermentation conditions or guide genetic modifications in a microbial host to optimize the yield and purity of its production and thus create opportunities for targeted postbiotic treatments [17,121,122].

## 8. Challenges and Future Outlook

While artificial intelligence and machine learning are undeniably transforming probiotic R&D, their full potential is far from being without significant hurdles. Overcoming these is of the highest priority for the sector to actually progress from promise to widespread, substantial clinical and commercial realities.

One of the main challenges is data challenges. The success of AI/machine learning models is inherently based on the quality and quantity of data they learn from, and this suggests that it is highly dependent on the data we feed it. Microbiome and probiotic research generate highly heterogeneous data—ranging from raw sequencing reads and high-complexity metabolomic profiles to diverse clinical outcomes and multifaceted lifestyle factors. It is critical but difficult to standardize data collection protocols across studies, maintain strong data curation, and make such massive datasets readily accessible while maintaining patient confidentiality. Without standardized, high-quality, and interoperable data, AI models risk perpetuating bias or making unreliable predictions, which will render the generation of truly robust individualized probiotic therapies impossible.

Another essential challenge is model interpretability and causality. Most powerful AI models, particularly deep learning networks, are “black boxes,” performing good predictions but not necessarily explaining why a particular decision or prediction was made. Within the biomedical and biological context, the underlying cause is as valuable as the prediction. Crossing correlation to test for causal relationships between probiotic interventions, shifts in microbiome, and host health outcomes is essential for regulatory clearance and clinician acceptance. Any future studies of AI/ML use in this area must prioritize the development of more explainable models and integrating them with experimental verification to uncover the underlying biological cause of their predictions.

Furthermore, commercialization and regulatory pathways are complex in themselves. The rapid acceleration of AI-augmented discovery, especially for genetically altered or highly personalized probiotics, has a tendency to outpace existing regulatory frameworks. Clear protocols must be developed for the regulation of AI-developed strains, novel forms, and tailored treatment suggestions. Modern regulatory networks, along with the significant capital required for the mass production and clinical trials, will be a leading force behind the commercialization of future probiotics.

Ethical considerations also need prime focus. With more sensitive personal health data being employed in customized probiotic intervention, privacy and data security become an utmost concern. Moreover, the field needs to proactively deal with social and ethical issues, including equitable access to high-tech personalized care, and manage public perception for trusted acceptance of AI-driven microbial treatments. Responsible innovation will unlock these massive technologies for all segments of society without exacerbating prevailing health disparities.

Lastly, the successful integration of AI/ML into probiotic research calls for profound interdisciplinarity. It will be essential to overcome the gap between bioinformaticians and microbiologists, and AI/ML researchers, clinical scientists, and social scientists as well. Only such a synergy will be required to design robust experiments, build advanced computational algorithms, interpret complex biological outcomes, and convert these into useful, safe, and cost-effective probiotic products.

## 9. Conclusions

The journey through the multifaceted world of probiotics, from their initial discovery to their present applications across a range of health scenarios, reveals a field poised for profound transformation. The inherent complexity of the human microbiome and the strain-specificity of probiotic action have long been great barriers, limiting the widespread and consistent success of these microbial therapies. But as this critique has carefully delineated, the advent of AI and ML has not only supported traditional probiotic studies; it has actually transformed its course, ushering in an age of unprecedented precision and creativity.

AI/ML’s disruptive power crosses the entire probiotic pipeline. From the initial discovery of novel probiotic strains through intelligent metagenomic prospecting and predictive trait scanning, to the optimization of their formulation and industrial production through AI-based fermentation management and stability prediction, all these technologies are streamlining every step. Above all, AI/ML is now enabling a more scientific understanding of how probiotics function, peeling back complex host–microbe relationships and defining distinct molecular mechanisms of action that were previously obscured by the sheer volume of biological information. That fundamental knowledge will be necessary to translate from empirical observation to rational design.

The true paradigm shift lies with the possibility of individualized probiotic therapy. With an integration of a patient’s own omics data—their genomics, microbiome type, metabolome, and habits—AI/ML can generate highly personalized probiotic prescriptions. This allows for prediction of probiotic efficacy for a specific condition, so interventions are tailored to maximize positive outcomes in each patient, rather than a “one-size-fits-all” approach. This level of personalization, once a dream on the horizon, is now rapidly becoming an actual reality, with AI being the force behind this revolution in precision medicine.

With advanced biotechnologies, there is a convergence of AI/ML leading to next-generation probiotics that are not only more potent but also smart. There is now a shift from mere beneficial microbes being isolated to the actual engineering of them with better functionalities as well as new metabolic pathways in the strains and fine-tuning the genetic architecture through more advanced tools like CRISPR-Cas. Besides individual strains, AI is refining synbiotic and postbiotic blends into high-performance combinations with specific, targeted health advantages. The fast-growing pace of discovery, supported by AI-enabled automation and robotics, complemented by a critical turning point towards sustainable feedstocks, has the prospect to render high-quality, sustainable probiotics broadly accessible.

Fundamentally, human–microbe destiny is being made actively by intelligent technologies. AI/ML is no tool; it is a collaborator, a creator, and a predictor in the endeavor to actualize the full therapeutic potential of the microbial universe. As we continue to develop these intelligent systems and synchronize them with bioscience, the world is on the verge of an era where probiotics will transform from general health supplements to customized, tailor-made therapeutics that will facilitate a healthier, greener, and truly bio-centric economy. This collaboration of human intelligence and artificial intelligence will certainly open unprecedented avenues to the preservation of health and the fight against disease through the brilliant manipulation of our microbial allies.

This review shows how artificial intelligence (AI) and machine learning (ML) are not complementary technologies but the prime drivers of a paradigm shift in probiotic research. By overcoming the inherent difficulty of multi-omics data and the limitations of the traditional “one-size-fits-all” model, these technologies have resulted in the development of targeted and personalized probiotic products. Specifically, AI/ML has yielded three major breakthrough outputs to the field: (1) facilitation of novel strain discovery by rapidly processing vast genomic and phenotypic data to select strains with desired bioactivity; (2) yield and stability enhancement through optimization of fermentation and formulation parameters to maximize probiotic robustness in the harsh intestinal setting; and (3) breaking resistant MoA deciphering by network analysis and dimensionality reduction of host transcriptomics/proteomics data to determine decisive molecular checkpoints, e.g., inflammatory pathways.

The immediate promise of this field is in the transition towards systematic and predictive modeling. This vision is achieved by creating AI/ML-enabled Digital Gut Twins, sophisticated computational models that can systemically and holistically mimic the effect of various probiotics on entire physiological axes, with the most important one being the gut–brain axis. This in-depth capability to actually forecast outcomes allows for an unprecedented reduction in the need for first animal and clinical trials, maximizing the R&D pipeline and allowing scientists to concentrate on only the very best interventions.

Successful application of such vast human body modeling remains, however, a daunting challenge. This is due to the necessity to integrate dynamic, multi-scale biological knowledge and to represent the intricate, non-linear behavior of organ systems. Despite this sophistication, the mission given to AI/ML in this situation is to verify probiotic effectiveness and direct interventions away from empirical validation onto precision medicine. The trajectory that AI has been on in this instance only confirms that future probiotic therapies will be scientifically rigorous, highly individualized, and strategically designed based on an individual’s individual biological blueprint.

## Figures and Tables

**Table 1:** A summary of AI/ML in Probiotic Strain Identification and Discovery.

AI/ML in Probiotic Strain Identification and Discovery	
**AI/ML in Probiotic Strain Identification and Discovery**	Metagenomic Sequencing of Environmental and Host-Associated Microbiomes [39,54,55]
	Large-scale Genomic Analysis of Known Microbial Collections [17,56]
	Deep Learning for Trait Prediction [57,58]
**Probiotic Trait Prediction Using Predictive Modeling**	Machine Learning Models that Classify Strains According to Desired Traits [61,62]
	High-Throughput Phenotyping Coupled with ML [63,64]
**Identifying Functional Genes and Metabolites**	AI-Driven Annotation of Novel Genes [66]
	Machine Learning for Identifying Novel Bioactive Metabolites [66]

**Table 2:** A summary of the main application areas of artificial intelligence and machine learning in probiotic science.

AI/ML for Understanding Probiotic Mechanisms of Action	Applications
**Deconstructing the Host–Microbe and Microbe–Microbe Interactions**	Predicting how a new probiotic strain integrates into the existing network, identifying keystone species, and understanding how probiotics compete or cooperate with other microbes [17,90,94].
**Predicting Impact on Host Physiology**	Predicting the probiotic’s effect on inflammatory pathways or neurotransmitter production. Also, identifying key bioactive metabolites (such as short-chain fatty acids) that are directly linked to health benefits [17,41,95,96].
**Modeling Microbiome Dynamics Under Probiotic Intervention**	Predicting the engraftment of probiotics in the gut, evaluating their impact on the stability of the resident microbiome, and providing personalized recommendations for probiotics [19,99,100,101].

## Data Availability

No new data were created or analyzed in this study. Data sharing is not applicable to this article.
